# A normative theory of luck

**DOI:** 10.3389/fpsyg.2023.1157527

**Published:** 2023-11-10

**Authors:** Chengwei Liu, Chia-Jung Tsay

**Affiliations:** ^1^European School of Management and Technology, Berlin, Germany; ^2^Department of Management and Human Resources, University of Wisconsin-Madison, Madison, WI, United States; ^3^Organisations and Innovation, University College London, London, United Kingdom

**Keywords:** luck, chance models, attribution biases, behavioral strategy, the Carnegie school, Matthew effect, simulation

## Abstract

Psychologists have identified heuristics and biases that can cause people to make assumptions about factors that contribute to the success of individuals and firms, whose outcomes may have actually resulted primarily from randomness. Yet the interpretation of these biases becomes ambiguous when they represent reasonable cognitive shortcuts that offer certain advantages. This paper addresses this ambiguity by presenting four versions (weak, semi-weak, semi-strong, strong) of a normative theory of luck that integrates insights from psychology with the chance model approach to predict the circumstances under which performance non-monotonicity occurs: higher performance may not only indicate greater luck, but also lower expected merit or quality. The semi-strong version is illustrated by examining the decoupling of citations of academic publications and their impact, illuminating when higher citations indicate lower quality. We conclude by discussing the broader implications of a normative theory of luck, emphasizing strategies to address situations where people mistake luck for skill.

## Introduction

Success attracts our attention. Yet research from psychology has demonstrated many attribution biases that arise when we evaluate success (Ross and Nisbett, 1991). For example, the outcome bias suggests that people tend to judge the quality of a decision based on its outcome instead of its process (Baron and Hershey, 1988), even when good decisions can lead to poor outcomes, and vice versa, due to unforeseeable circumstances (Barney, 1997). This tendency may be exacerbated by the hindsight bias (Fischhoff, 1975). Even when success can largely be attributed to luck, there is often something unique in a person or organization’s history that can be cited to construct a plausible, yet untrue, narrative to support why the successful deserve the glory and reward associated with their successes—and accordingly, the stigmatization associated with their subsequent downfalls (Rosenzweig, 2007; March, 2012; Frank, 2016; Pluchino et al., 2018). A shared insight of these findings is that people tend to mistake luck for skill when evaluating achievement (Taleb, 2001; Makridakis et al., 2009; Kahneman, 2011).

However, the interpretation of these biases can become ambiguous when biases represent reasonable shortcuts that offer competitive or evolutionary advantages (Gigerenzer and Goldstein, 1996; March, 2006). For example, one of the most primitive heuristics among humans is to imitate the most successful (Richerson and Boyd, 2005; Rendell et al., 2010). Although this heuristic is subject to a strong outcome bias, the learning outcome may be adaptive in the sense that the most successful may be luckier than, but also superior to, the less successful. The motivational effect of holding the successful up as role models also generates beneficial exploration (due to imprecise copying) for the community (Dosi and Lovallo, 1997; Posen et al., 2013; Liu, 2020). The net effect of learning from the successful can be positive, implying that biases, such as misattributions of luck, can persist because they produce outcomes that are “better than rational” (Cosmides and Tooby, 1994).

Yet adaptive heuristics can become maladaptive due to mismatches: applications of heuristics to novel contexts where the underlying mechanisms appear similar but are qualitatively different. For example, learning from the most successful may no longer make sense in modern contexts where social or competitive mechanisms (such as rich-get-richer or winner-takes-it-all dynamics) can augment the impact of luck to such an extent that the more successful can even be inferior to their lower-performing counterpart (Levy, 2003; Denrell and Liu, 2012; Frank, 2016). Imitating the successful can be misleading and detrimental. To illustrate, a recent study shows that earnings can be negatively correlated with cognitive abilities beyond a certain threshold (Keuschnigg et al., 2023). That is, the highest earners have lower average cognitive abilities than their lower-earning counterparts. Their exceptional success—earnings several orders of magnitude higher than the rest—reflects more how factors beyond their control (e.g., inherited socio-economic status) were favorably reinforced rather than being a reliable indicator of their exceptional merit. Imitating such “successes” would thus lead to disappointment: Even if one could strive to replicate everything they did, one would not be able to replicate their luck.

To identify when learning from the “successful” is misleading, we present a normative theory of luck that predicts which performance range may be a less reliable indicator of merit and, in turn, entails fewer opportunities for learning. This theory draws insights from both chance models and psychology. Chance models aim to develop a theoretical mechanism that explains empirical regularities through the interaction of randomness and structured environments (Denrell et al., 2015; Liu and Tsay, 2021). In the context of learning and evaluation, chance models help predict when performance non-monotonicity occurs—that is, when higher performance actually predicts lower expected merit. Non-monotonicity violates the conditions for more typical evaluation heuristics, such that higher performers are, on average, superior (Milgrom, 1981), and instead predicts detrimental learning or imitation. Research from psychology further develop the implications: How attribution tendencies, such as the outcome bias, halo effect, and fundamental attribution error, predict how people may be rewarded or punished for performance that actually stems from luck. Normative implications can then be developed to help remedy biases in evaluating employees or to arbitrage the resources mispriced by rivals due to their biases (Liu et al., 2017; Denrell et al., 2019; Liu, 2020, 2021a,b).

The application of a normative theory of luck is illustrated by exploring a question that is relevant to many academics: When do high citations of academic papers reflect superior merit or impact? We first use an exploratory survey to illustrate that more highly cited papers are not necessarily more impactful: The number of citations articles received and the votes they received for impact in the survey can be negatively correlated for moderately highly cited papers, particularly for papers published in the field of management. Integrating theory across literatures with the patterns suggested by our exploratory survey, we develop a chance model to explore the mechanism for this non-monotonicity—a middle dip in the association between outcome and merit—and offer additional analyses. Our results suggest that moderately high outcomes entail greater uncertainty when a strong reinforcement mechanism is present; this level of “success” is most likely to be achieved by agents with mediocre merit, combined with a strong reinforcement of early luck (e.g., the fame of one of the authors or the popularity or timeliness of the topic). Agents with outcomes just below the middle dip are likely to have superior merit but early bad luck, bounding the eventual outcome they can achieve. The negative correlation between citation and impact point to important implications for how citation measures should be used in the evaluation of academics.

The paper is structured as follows: We first offer a primer of chance models that provide the foundation of a normative theory of luck. We then report when highly cited papers predict greater impact, as measured by our exploratory survey results from management and psychology academics. Prompted by survey patterns, we develop a chance model to reproduce the empirical patterns we found, thus providing a possible explanation for them. While prior chance models predict that top performers are likely the luckiest and associated with lower expected merit, our results produce a novel version of the normative theory of luck: The performance non-monotonicity occurs not at the extremes, but instead in the middle range. We conclude by discussing the implications of a normative theory of luck, including how to remedy the bias (e.g., through random allocation) and how to arbitrage the resources mispriced by rivals (e.g., searching for “hidden gem” papers with citations below certain thresholds).

## Theoretical foundation

### A primer on chance models

Luck—the impacts brought by chance events—is often cited as a factor relevant to important phenomena in the social sciences (Liu, 2020). Some see luck as a solution; for example, political scientists posit that random selection does not discriminate and can thus help resolve political deadlock by offering fairer results to competing parties (Carson et al., 1999; Stone, 2011). On the other hand, economists view luck as “noise”; for example, even when unexpected shocks create mispriced assets, market inefficiencies are fleeting, as they tend to be arbitraged away quickly by rational traders (Fama, 1970).

Other aspects of luck have also intrigued social scientists. For example, behavioral economics and finance researchers have shown that market inefficiencies can persist if decision makers are unable to self-correct, such as when investors make misguided conclusions about randomness (Shleifer and Vishny, 1997; Taleb, 2001; Thaler, 2015). Similarly, sociologists have explored the role of luck on status in society, such as how reinforcing mechanisms (e.g., the “Matthew effect”^1^) could contribute to the accumulation of socioeconomic inequalities (Merton, 1968; Lynn et al., 2009; Sauder, 2020). Finally, psychologists have studied luck as an attribution factor for decades (Kelley, 1971; Baron and Hershey, 1988; Hewstone, 1989), and later research highlights how luck attributions impact self-reflection, identity construction, and ethical judgment (Kahneman and Tversky, 1982; Kahneman and Miller, 1986; Roese and Olson, 1995; Teigen, 2005).

However, few of these perspectives study luck as *the* explanation for behavioral or complex phenomena. To illustrate this unique perspective that places luck in a more central role, consider a chance model application in psychology: The subadditivity in probability judgments, which states that the probabilities of mutually exclusive events cannot exceed one. Yet, past research in psychology shows that when individuals are asked to evaluate the probabilities of such events, their answers often sum to more than one (Dougherty and Hunter, 2003). This phenomenon of subadditivity has been extensively studied by experimental researchers, who typically attribute it to systematic cognitive biases (Fox et al., 1996). For instance, one argument suggests that detailed descriptions of events evoke multiple associations, leading to overestimation (Tversky and Koehler, 1994). Here, a chance model proposes a more parsimonious explanation by assuming unbiased but noisy probability judgments (Bearden et al., 2007). In this model, probability judgments are unbiased on average, but subject to random noise. When an individual evaluates the probabilities of several mutually exclusive events, the average probability of each event must be relatively small, since they sum to one. Consequently, even unbiased but noisy estimates, due to random variability, will tend to result in overestimation. This is because when the true probability is close to zero, there is a “floor effect.” For example, if the correct probability is 0.1, the event can only be underestimated by at most 0.1, but can be overestimated by a larger magnitude. The behavioral regularity of subadditivity may be explained by a chance model without assuming cognitive biases.

Another example of an application of the chance model comes from organization science. Consider the empirical regularity of age dependence in failure rates: The failure rates first increase with firm age and then decrease (Freeman et al., 1983). The assumed explanation is a liability of newness, combined with learning: Young firms with little experience are more likely to fail, and survivors who learn from past blunders become more viable over time. However, a random walk process with an absorbing lower bound can reproduce this empirical regularity without assuming a learning effect or differences in capabilities among firms (Levinthal, 1991). The initial increase may be attributed primarily to early bad luck rather than to the liability of newness; firms that happen to receive negative shocks early on are forced to exit. The later decrease in failure rates may be attributed primarily to early good luck rather than to learning or improvement. Firms that did not fail early on are likely to accumulate sizable resources that will keep them further away from the lower bound, making them less likely to fail over time. Thus, a chance model can provide an alternative explanation for the age dependence in failure rates (Denrell et al., 2015).

The chance model approach—the perspective of seeing randomness operating in a structured environment as the explanation for behavioral or complex phenomena—is built on the insights of James March, one of the founders of the Carnegie Perspective of Organizational Learning and Decision-making. The Carnegie Perspective was established from three classic books in organization science (Simon, 1947; March and Simon, 1958; Cyert and March, 1963). The founders’ shared premise was to study organizations by understanding how boundedly rational actors make decisions with behaviorally plausible mechanisms under constraints in communication, coordination, and structure (Gavetti et al., 2007). Notably, luck was not a central construct in the Carnegie Perspective until March developed various “chance models” with his coauthors (for a review, see Liu and Tsay, 2021).

A classic example of a “chance model” is the garbage can model of organizational choice (Cohen et al., 1972), which highlights how disconnected problems, solutions, participations, and choice opportunities can be lumped together coincidentally in decision making and behavior instead of through rational design or the logic of consequence. “Luck” was added to the list of “behaviorally plausible mechanisms” in the sense that the aggregation of intentional actions could nevertheless appear non-systematic, and vice versa. A key takeaway is that luck should be considered as a default explanation for complex behavioral or organizational phenomena until strong counterevidence emerges (Denrell et al., 2015). Believing otherwise increases the risk of being misled by randomness and suffering from the illusion of control (Langer, 1975; Taleb, 2001; Liu and de Rond, 2016).

### Toward a normative theory of luck

Lay theories of luck tend to be normative but unreliable (e.g., choosing lucky numbers increases the chances of winning), whereas academic theories of luck tend to descriptive (e.g., events beyond our control changes the course of history) and highlight their subjective nature (e.g., luck is in the eye of the beholder; an unlucky event can be a blessing in disguise with additional knowledge). We argue that, from a learning point of view per the Carnegie Perspective, a normative theory of luck is possible. That is, one could formalize the conditions under which a particular performance range may be more subject to random processes and a less reliable target for learning and aspirational imitation.

In particular, a normative theory of luck focuses on circumstances under which success can be a misleading indicator of merit—that is, when higher performance fails to indicate superior merit or set a good benchmark for learning and imitation. It requires the application of chance models to demonstrate when performance non-monotonicity occurs: Higher performances may indicate not only greater luck, as prior studies suggest (Kahneman, 2011; Mauboussin, 2012; Frank, 2016), but also lower expected merit. This is important because of the long tradition of learning from the successful across cultures in human history (Richerson and Boyd, 2005). “Successes” are usually compressed to a single dimension for the ease of learning and performance appraisals, such as returns on assets when comparing firm performance, wealth or income when comparing people, and number of publications and their citations when comparing academics. The heuristic of learning from the most successful is predicated on the assumption that the more successful are superior, on average, and thus are better role models. Chance models provide a critical lens to evaluate when learning from the successful can be misleading. Here, we briefly review three chance models before building on them to specify a normative theory of luck.

#### The March 1977 model: the almost random careers of senior executives

Past research in psychology has demonstrated that there is a tendency to give credit to the individual instead of the circumstances (Ross and Nisbett, 1991). March developed a series of “chance models” to challenge this assumption by showing how randomness and situational factors play more important roles in outcomes (March and March, 1977, 1978). The mechanism builds on a natural consequence of selection: variation reduction. The average skill of each round of surviving candidates increases over time because the least skilled employees are sorted out. However, an important side effect of selection is often neglected: the reduction of diversity (specific to the variance in skill) among survivors. This reduction effect, also known as the “paradox of skill,” holds whenever the same selection criteria (e.g., having a college degree or not; publishing a certain number of academic papers; reaching a sales target) are applied to all candidates (Mauboussin, 2012; Page, 2017). The implication is that the eventual survivors—those who passed multiple rounds of selections in an organization or system—are very skilled, but the differences among them are very small, making the survivors increasingly indistinguishable from those selected out (Denrell et al., 2017). The results, based on analyses of a set of Wisconsin superintendents’ data, largely supported the predictions: Transition probabilities (e.g., being promoted or fired in this school system) did not vary by individuals but instead were fixed. This suggests that career trajectories may have been approximated by chance fluctuations rather than by any individual-level characteristics of the superintendents, a phenomenon summarized aptly by the title of the paper, “Almost Random Careers” (March and March, 1977). The implication is that successful career outcomes among these superintendents may not have been a reliable indicator of superior merit but may instead have reflected the superintendents being at the right place and right time.

#### The March 1991 model: winners are overrated

One of the most prominent articles in organization science highlights a tension that arises when organizations try to balance exploration and exploitation (March, 1991), and offers a chance model that explores this dilemma in the context of competition (Model 2). As illustration, consider numerous firms competing to obtain the highest performance in an industry. Their performance draws from normal distributions with varying means and variances. This work showed that exploitation (defined as a pursuit of higher mean performance) becomes increasingly irrelevant when the number of competitors increases. In fact, only exploration (defined as a pursuit of variance in performance) matters when the number of competitors approaches infinity: The top performer is likely the firm that has the highest variance, regardless of its mean performance. The problem is that introducing competition becomes counterproductive: The winning firm is not necessarily better than others, and this mechanism introduces adverse selection. That is, firms with a low mean performance are motivated to take excessive risk in order to enhance the chance of finishing first, which they could not have otherwise achieved, as illustrated by forecasts made by Wall Street analysts (Denrell and Fang, 2010). The implication is that success under intense competition may not be a reliable indicator of merit but may instead reflect high variance and excessive risk-taking.

#### The Denrell and Liu 2012 model: the most successful may be worse

The most successful performer may be luckier than others, but learning from them may still be sensible if they are, on average, superior to the rest. Denrell and Liu (2012) developed a chance model to demonstrate when being a top performer indicates not only a high degree of luck, but inferior expected merit. Their model builds on the two earlier March models and generates novel predictions. March’s 1977 model implies that the differences among agents who survive multiple rounds of competitive selections are small. March’s 1991 model implies that when competition is intense, variance is important in determining the outcome. Denrell and Liu’s 2012 model assumes agents’ performance depend on both their merit and the strength of the reinforcing mechanism. Drawing insights from March’s chance models, the distribution of merit is more compressed than that of reinforcing mechanisms. The implication is that trivial initial differences due to randomness can be augmented by a strong reinforcing mechanism, overwhelming the importance of merit and decoupling the typical association between merit and outcomes. The decoupling can be so strong that top performers can be associated with the strongest reinforcing mechanism (and benefit from “boosted” luck), without necessarily achieving the highest merit. The less exceptional performers, or “the second best,” thus tend to have both the highest expected merit and highest expected future performance. Denrell and Liu’s 2012 chance model prediction is also illustrated by the income-cognitive ability association mentioned earlier: Individuals with high but not top earnings have the highest level of cognitive abilities.

Consider an example in the music industry using the association of consecutive performances. If a musician has a Top 20 hit, should we infer exceptional talent from their success? Liu’s (2021c) analysis of 8,297 acts in the US Billboard100 from 1980 to 2008 would suggest not. Music-label executives should instead try to sign those who reach positions between 22 and 30, the “second best” in the charts.

One example is the Korean performing artist PSY, whose “Gangnam Style” music video went viral beyond anyone’s foresight. Since such an outcome involved exceptional luck—early luck combined with a strong word-of-mouth effect—PSY’s success was unsustainable. In fact, artists charting in the Top 20 will likely see their next single achieve no higher than between 40 and 45 on average; they regress disproportionally more to the mean than their lower-performing counterparts. The exceptionally successful cannot replicate their exceptional luck. Those charting between 22 and 30, meanwhile, have the highest predicted future rank for their next single. Their less exceptional performance suggests that their successes depended less on luck, making their performance a more reliable predictor of their merit and future performance. The implication is that success can be a misleading indicator of merit, reflecting exceptional luck and circumstances that are not replicable or sustainable.

#### Three existing and one emerging versions of the normative theory of luck

A normative theory of luck builds on the three chance models reviewed in the last section. Table 1 provides a summary. The 1977 March model can be considered a weak version: Success is an unreliable indicator of merit because agents are all highly skilled due to competitive selection. The more successful are more likely to reflect their luck instead of superior merit. The expected merit likely plateaus beyond a certain level of performance. The 1991 March model can be considered a semi-weak version of luck: Success is an unreliable indicator of merit because winning under intense competition requires not merit, but excessive risk taking. The more successful are more likely to reflect their risk-taking, producing favorable outcomes by chance, instead of superior strategy or foresight in competition. The 1991 March setup still predicts that the expected merit likely plateaus beyond a certain threshold, but an additional inference about the level of risk-taking can be made. In contrast, the Denrell and Liu 2012 model can be considered a strong version: Success can be a negative indicator of merit in the absence of competition because exceptional success tends to occur in exceptional circumstances. The most successful are likely to obtain their outcomes in contexts with a strong reinforcement mechanism. However, in such contexts, early luck can overwhelm merit, generating a negative correlation between success and merit at the highest performance range.

**Table 1:** A summary table of the different versions of the normative theory of luck.

Normative theory of luck	Key reference	Key mechanisms	Empirical illustrations	Stylized predictions
Weak version	March and March (1977)	Individuals who passed through multiple rounds of selections in a system are similarly competent, meaning their performance differences are uninformative about their merit or competence.	Wisconsin superintendents’ career movement (March and March, 1977)	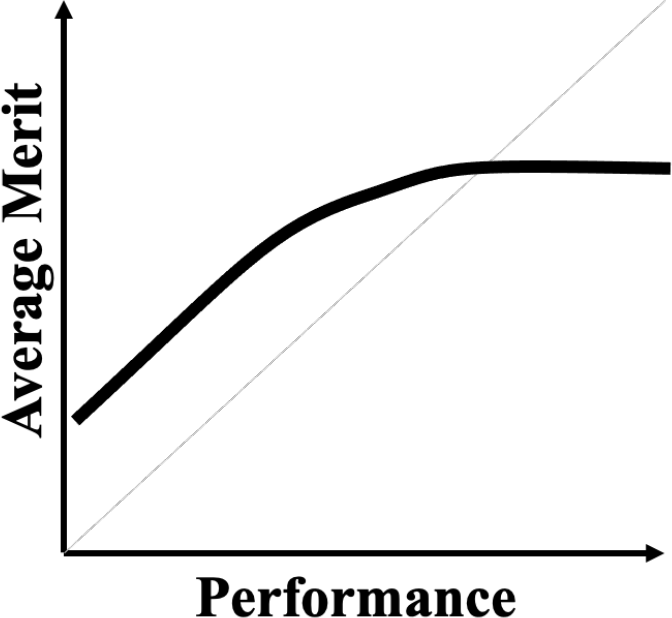
Semi-weak version	March (1991)	When outcomes are winner-takes-it-all and driven by both merit and risk taking, top performers are likely the ones that take extreme risk, regardless of their merit.	Forecasts made by Wall Street analysts (Denrell and Fang, 2010)	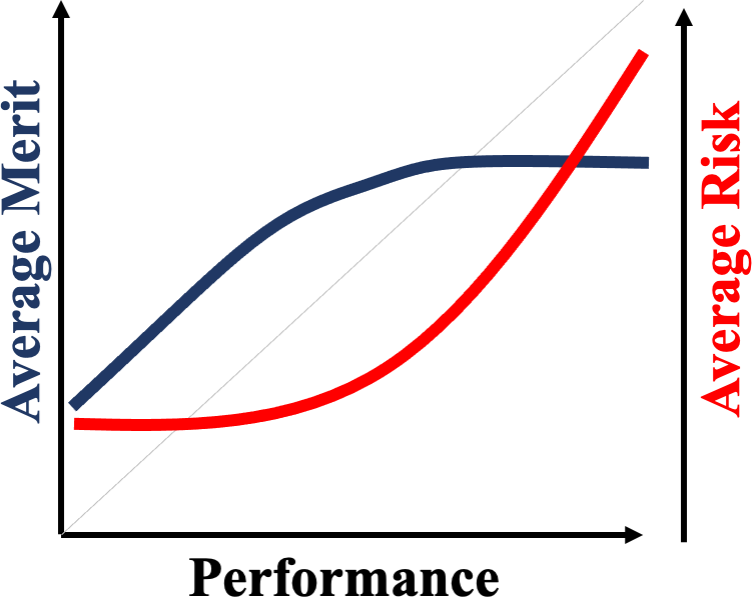
Semi-strong version	Current paper; Denrell and Liu (2021)	When performance depends on both merit and past performance and the reinforcing mechanism is strong but fixed for all, an N-shaped performance non-monotonicity occurs.	The citation-impact association (current paper); The movie sales-rating association (Denrell and Liu, 2021)	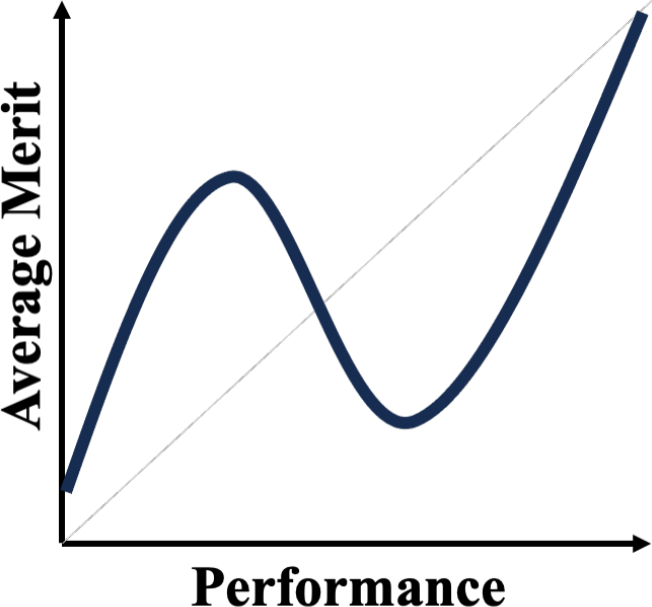
Strong version	Denrell and Liu (2012)	When performance depends on both merit and past performance, yet the distribution of merit is less variable than that of the reinforcing mechanisms, a S-shaped performance non-monotonicity occurs.	The income-cognitive ability association (Keuschnigg et al., 2023); The Billboard Hot 100 analysis (Liu, 2021c)	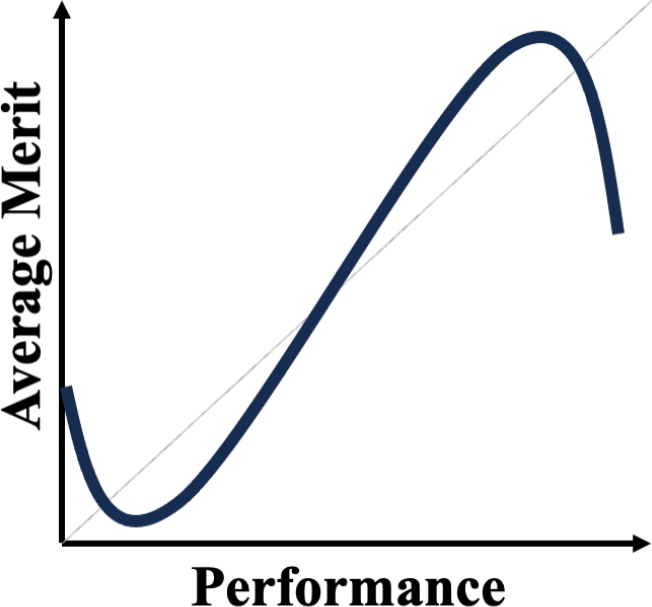

Another common characteristic among the three chance models is that they all connect to empirical regularities: the almost random career of Wisconsin superintendents; wild-card forecasts by Wall Street analysts who predicted the next big thing; the income-cognitive abilities association; top-ranked musicians whose performance subsequently regressed to below average. These events all challenge the usual assumption that higher performers are superior. The relationship between success and merit above a certain level of performance can flatten (as the 1977 and 1991 March models predict) or become negatively associated (as the Denrell and Liu model predicts). The fifth column in Table 1 illustrates their different implications for inferring merit from different performance levels. The strong version of the normative theory of luck presents performance non-monotonicity and hence rank reversal: Higher performers can be worse than their lower-performing counterparts, implying a systematic failure when applying the usual heuristic of learning from the most successful.

The existing versions of the normal theory of luck also inspire the recombination and exploration of new possibilities. One underexplored assumption is the situation in which the reinforcing mechanism is strong but does not vary across individuals. The 1991 March model and the Denrell and Liu 2012 model assume that the reinforcement mechanism is not only strong, but also varies across individuals. Yet in many contexts, individuals share the same level of reinforcing mechanism. For example, in academia, the Matthew Effect may be both strong and different across fields. But within the same field, academics are subject to the same level of the Matthew Effect. The existing chance models do not provide a clear prediction of what the association between performance and merit may look like.

In the next section, our empirical exploration examines this possibility. To preview our finding, performance non-monotonicity occurs—not at the top level of success, as the three models reviewed in this section suggest, but at moderately high level of success. That is, the results show a N-shaped pattern: For a given academic field (such as management) where the reinforcement mechanism is strong but does not vary, expected impact first increases with citations, then decreases in the middle range, and then increases again for the most highly cited papers. This pattern is stronger for papers published in management than in psychology. A chance model is then developed to unpack the underlying mechanism, particularly how it differs from the Denrell and Liu 2012 model. This illustration of a novel performance non-monotonicity enriches the normative theory of luck by providing a “semi-strong version”—strong because it generates performance non-monotonicity and rank reversal—and offering important implications for performance evaluations in academia and beyond.

## When highly cited papers are “worse”

To illustrate a normative theory of luck, we build on the latest developments of chance models (Denrell and Liu, 2021; Liu and Tsay, 2021) and apply their implications to a question relevant to many academics: When would a high level of citations of academic papers reflect superior merit or research impact? Many practices and policies in academia, such as recruitment, promotion, and grant allocation, assume that highly cited papers tend to be associated with higher-impact research (Kaplan, 1965; Small, 2004; Cronin, 2005). However, work on the Matthew Effect suggests that increasing recognition, including citations of papers, does not necessarily imply higher-impact research, but rather good fortune combined with strong reinforcing processes (Merton, 1968; Baum, 2011). For example, papers that are published by prominent authors or on timely topics may attract more attention and, in turn, elicit more initial citations than other papers of similar or superior merit or potential impact (Liu et al., 2017). The initial difference in citations can be augmented to such an extent that the eventual citation count decouples from expected merit or impact, and generates a non-monotonicity (Lynn et al., 2009; Denrell and Liu, 2012, 2021); more highly cited papers may even be associated with lower expected impact. Using exploratory survey results from academics, we empirically examine this theoretical prediction in the context of academia by measuring the association between citations and impact.

Notably, we are not arguing that highly cited papers always indicate lower expected impact or merit. Instead, we investigate when citations may be a less reliable indicator of impact or merit. Measuring the merit of a paper is very challenging, and many people simply use citations as a proxy for a paper’s expected impact. However, as discussed in the previous section, reinforcing mechanisms can sometimes decouple outcomes (such as citation count) from merit (such as papers’ counterfactual impact without the influence of the Matthew Effect) to such an extent that outcomes and expected merit may even become negatively correlated (Denrell and Liu, 2012). One needs an alternative measure for merit to avoid the confounds that may emerge from a reliance on citations alone. To address this challenge, we conducted two exploratory surveys in which we asked academics in both management and psychology to vote for papers that they considered to offer higher impact. Motivated by the results suggested by the surveys, we then developed a chance model to account for the empirical patterns found, including differences between the two fields.

### Survey method and result

For the survey of management academics, we selected the 15 most prestigious management journals and the three all-time most-cited papers in each journal, which generated 45 seed articles. The journals were selected to provide an overlap between the 50-journal list developed by the *Financial Times* to calculate business school rankings and the list developed in Podsakoff et al.’s (2008) bibliometric analysis of management articles, including all management journals in the ISI Web of Knowledge database, which publishes the impact factors of journals. Only these 15 journals are considered top journals by both academics and practitioners. We then selected the top three most-cited papers in each of the 15 journals. For the survey of psychology academics, we selected 20 top psychology journals using a similar approach. More journals were included in psychology because it is a larger field than management and because more journals in psychology satisfy our selection criteria. The three most-cited papers in each of the 20 psychology journals were used as our seed articles for the survey, providing 60 seed articles.

**Figure 1:**
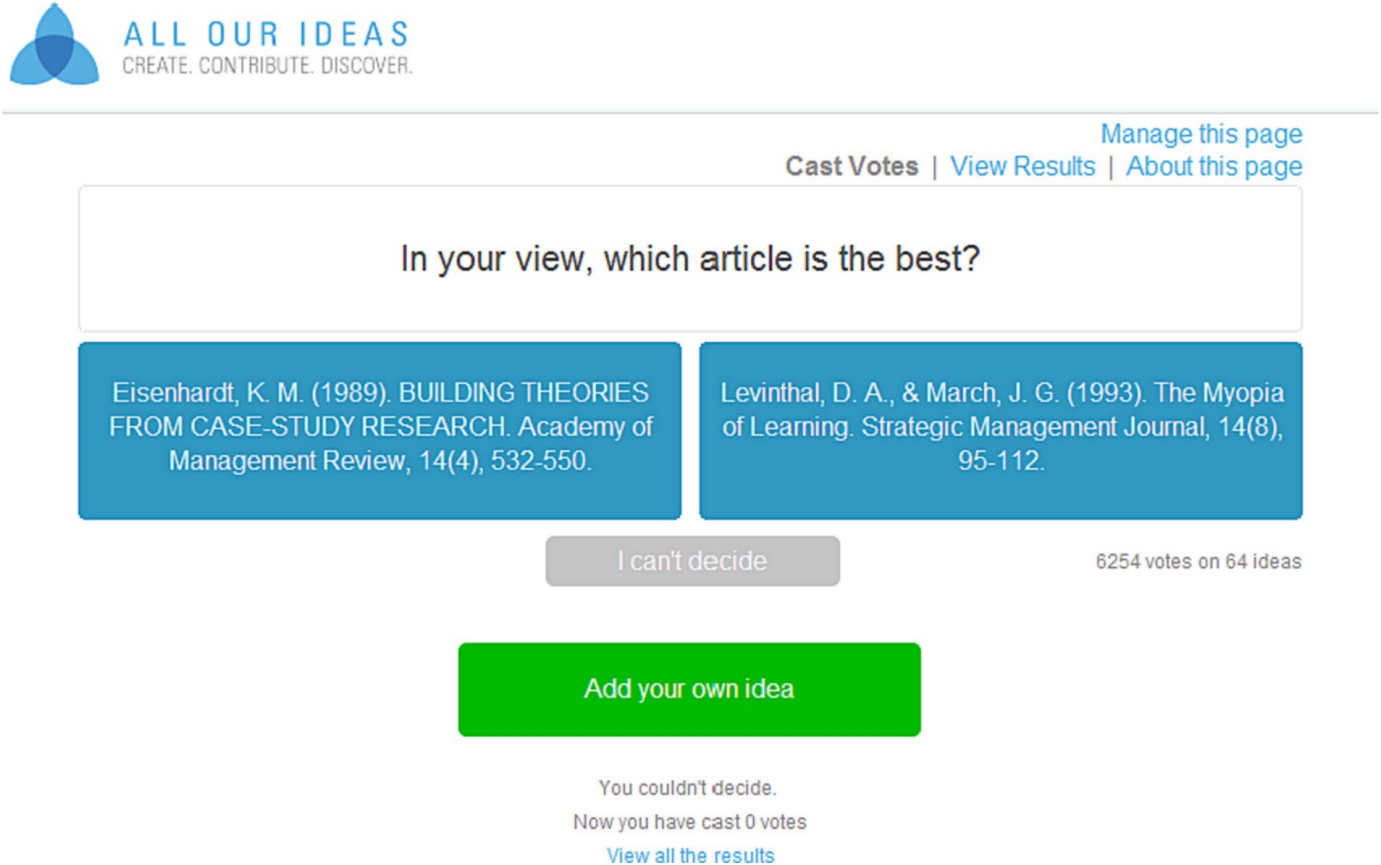
A screenshot of how the exploratory survey was conducted on the platform All Our Ideas.

We conducted our survey using All Our Ideas, a crowdsourcing platform developed by Matthew Salganik, which offered three unique features that aligned with our intended design. First, the platform implemented the survey as a pair-wise comparison. Participants saw two articles randomly drawn from the pool of all articles in each vote and were asked, “In your view, which article is the best?”^2^ Participants selected one of the two or chose “I cannot decide.” They voted as many times as they wished. Figure 1 provides an illustration of how the survey was run.

The second unique feature was that the platform allowed participants to add their input for seed articles. Thus, participants nominate preferred management/psychology articles to the pool, in addition to the seed articles, as long as the suggested articles were published in one of the selected journals. A pool of 64 ideas (i.e., papers) was generated for the management survey, which implies that 19 articles were added by participants. For the psychology survey, four articles were added by the participants, suggesting a final pool of 64 papers.^3^

The third feature of the platform was the algorithm developed to calculate a score measuring which of the ideas on the platform were most likely to win. A score of 80 for an idea (or paper, in this case) suggests that it has an 80% chance of being considered a better idea than a randomly chosen idea from the pool. Since we had two types of papers, seed papers and participant-added papers, we calculated the ranking of all ideas based on the scores representing the merit or impact of each paper.

We invited academics in management and psychology to participate in the survey by sending emails to lists managed by professional associations, including the Academy of Management and the American Psychology Association, and our professional networks. Appendix A documents the text of our invitation email. According to Google Analytics (a website traffic-tracking tool), for the management survey, we had 680 participants from 43 countries, who together cast 6,254 votes (each vote corresponding with one pairwise comparison, not including “I cannot decide” votes); for the psychology survey, we had a total of 943 participants from 27 countries, who together cast 3,524 votes.

We are interested in whether highly cited papers are viewed as higher in impact—that is, using our proxy for impact in the form of votes among academics. We analyzed an overall association between the citation count of articles and the average votes they received. If the number of citations is a good indicator of impact, the association between citations and expected quality should be a monotonically increasing function with strong positive correlation between the two variables. If the citation count is not a good indicator of merit, the correlation between citation and expected quality should be low, if not negative.

The results show that the associations between citation count and paper impact are positively correlated in both management (0.44) and psychology (0.37). Higher citation counts are associated with higher expected impact in both fields when both variables are included in a linear regression model. However, these initial analyses omit a more nuanced view of these associations and of the differences in patterns that emerged, which the next section details.

### Explaining the “middle dip” using a chance model

**Figure 2:**
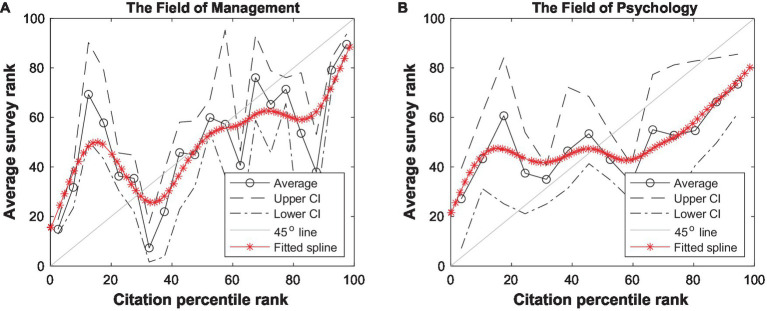
The association between papers’ citation count and their impact (as measured by survey rank) in the field of **(A)** management and **(B)** psychology.

To further examine the association between citation count and paper impact (using voting rank as a proxy), we first compute how papers’ average voting rank, based on our survey responses, vary with their citation counts and then fit a spine to the association. As Figure 2 shows, there is a notable difference in the degree of monotonicity between the fields of management and psychology. Both relationships are not strictly monotonic (as indicated by the best-fitted spline^4^), and the dip in the middle range (articles with 250–400 citations) is much more salient in management than in psychology.

Our exploratory survey results (see Figure 2) present an interesting empirical pattern: Highly cited papers do not necessarily receive more votes, particularly in the middle range of citations in management. In particular, across fields, for the moderately highly cited papers, the correlation between citation count and votes is weak. The association is almost flat in psychology and even becomes negative in management.

In this N-shaped or “middle dip” pattern, the expected value first increases with input, then decreases or flattens, and then increases again for high values of the input. There are many possible explanations for this pattern, such as sampling issues regarding participants or seed papers.^5^ We cannot examine these explanations directly due to limitations of the survey. However, we can use these exploratory survey results and the patterns they suggest to develop a chance model to examine one possible explanation: How initial recognition through citations, combined with the reinforcing mechanism, may generate the middle dip.

Our prediction, spurred by our survey results, is that when early citations are imperfect due to network effects, luck, or noise, a strong reinforcing mechanism (such as the Matthew Effect) can allow some papers to receive many citations despite having low impact—specifically, the papers that fall in the middle-dip region in management or the flattened region in psychology. In other words, the few papers that manage to get a cluster of initial citations may then be elevated to receiving more attention and more ensuing citations than comparable other papers. To reach top citation counts, however, papers need both high potential impact and good initial recognition (receiving early citations and then benefitting from the Matthew Effect), which would account for why the association between citation count and impact becomes strongly positive at the upper percentile ranks in both psychology and management.

To examine our proposed mechanism, consider a simple chance model where recognition (e.g., citing papers) is influenced by both merit or impact (e.g., a paper’s contribution to the literature without the influence of the Matthew Effect) and other agents’ choice behaviors (e.g., accumulated citation count thus far or strategic citations). Suppose there are *n* items, which can be products or services on recommendation systems or academic papers that can be cited by peers. The “quality” of item *i* is *q_i_*, where *q_i_* is drawn from a bell-shaped distribution between zero and one. “Quality” can represent the stable trait of a product or paper (Salganik et al., 2006), which we previously refer to in our exploratory surveys as impact, as operationalized by votes from academics. The appeal (*u*_*i*,t_) of item *i* in period *t* is *u_i,t_ = aq_i_ + bm_i,t − 1_*, where *m_i,t_* represents the choice proportion of item *i* from *t = 1* up to period *t − 1* (we set *m_i,0_ = 1/n*). The parameter *a* represents the weight allocated to quality, such as recommendation system users’ own judgment about an item’s quality. The parameter *b* represents the weight allocated to “past data,” such as the cumulative citation count of a paper or cumulated market share of items on the recommendation system. The probability that item *i* will be recognized by an agent (e.g., an author who chooses to cite one out of *n* papers) joining in period *t* follows the multinomial logistic choice model:


$$P_{i,t}=e^{u_{i,t}}/\overset{n}{\underset{j=1}{\sum }}e^{u_{j,t}}$$

**Figure 3:**
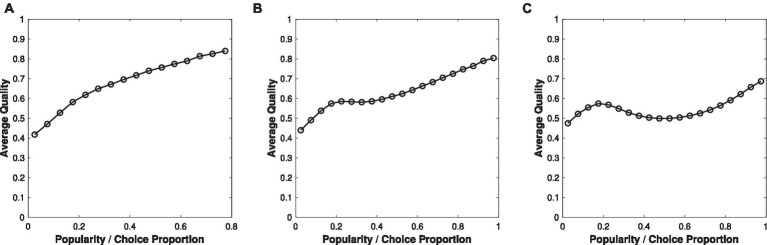
How average quality varies with recognition—proportion of item i being chosen at period t = 1,000 (i.e., after 1,000 participants made their choices) for three assumptions regarding the weight given to past popularity: **(A)** weak Matthew Effect, with *b* = 2; **(B)** moderate Matthew Effect with *b* = 3; **(C)** strong Matthew Effect with *b* = 4.

Figure 3 shows how average quality varies with the choice proportion obtained after 1,000 periods (i.e., as if 1,000 academics have made their citation choices) for different values of *b* when *n* = 10 (ten papers) and *a* = 1. A higher choice proportion is associated with higher average quality only when the weight on past data is not high, such as *b* = 2 (Figure 3A) or *b* = 3 (Figure 3B). When the weight on past data is high (such as *b* = 4, Figure 3C), outcomes can become a misleading indicator of quality. That is, there is a strong decoupling in the middle range. Consider academic citation counts: This means that papers with relatively low impact could gain moderately high citation counts if they were recognized early on. A strong Matthew Effect ensures many subsequent authors will cite these lucky papers, despite an absence of high impact or quality. In contrast, papers with high impact can get trapped with low citations when their early lack of recognition is augmented by the Matthew Effect, i.e., a poor-gets-poorer process. However, because parameter *a* is greater than zero, meaning that impact still plays a role in choice behaviors, low-impact papers would fail to receive endorsements from all citing academics, thereby limiting their highest possible citation counts. Only papers with top potential impact, combined with early recognition, would receive more global endorsements and achieve the highest ultimate recognition.

The decoupling is the strongest in the middle range when the reinforcement is strong (e.g., Figure 3C). Importantly, items with moderate quality (around 0.5, which represents the majority, assuming a bell-shaped distribution) are most sensitive to this decoupling: Early luck or recognition, instead of academic merit, is a strong predictor of the eventual outcomes to which such papers will be locked in. Outcomes may not reflect meritocratic processes if choice behaviors place too much weight on past success. More successes only strengthen, rather than correct for, any locked-in status and can create illusory predictive accuracy (e.g., such as the assumption that citation counts would be a reliable indicator of quality based on their continuous growth), generating a learning trap that is difficult to overcome (Liu, 2021c).

### Discussion

As Figure 3 shows, a simple chance model that assumes a stochastic process in choice behaviors, in the presence of a reinforcing mechanism, can reproduce the empirical patterns we found from the exploratory survey (see Figure 2). In particular, the pattern from psychology—a flattened association in the middle range—resembles the results when the reinforcement effect is moderate (*b* = 3). The pattern from management—a middle dip— resembles the results when the reinforcement effect is strong (*b* = 4). All else being equal, the strength in how early recognition or luck is reinforced may account for differences we found across fields in the association between citation count and quality.

Ideally, the association should be strongly positively correlated, as Figure 3A suggests (when *b* = 2, with a weak reinforcement effect). This is when using citation metrics as an input for performance appraisals would be reasonable: Highly cited papers indicate superior impact across the citation range. However, the association between citation count and impact becomes negative for moderately highly cited papers. Our results thus suggest that the practice of using citation metrics in performance appraisals may be problematic even in psychology, where highly cited papers are not necessarily better than less cited ones. Instead, highly cited papers may simply be lucky and actually of comparable impact, based on early recognition and the receipt of more attention than the works deserve. That is, high but not top citation counts are more likely to reflect initial luck instead of impact or merit. Their lower-cited counterparts may actually be more impressive, in the sense of receiving a decent number of citations (e.g., around 100–150), despite having early bad luck, in the form of a lack of initial recognition. Thus, the practice of using citation counts to reward academics can become misleading and introduce a lack of fairness, particularly for management academics.

**Figure 4:**
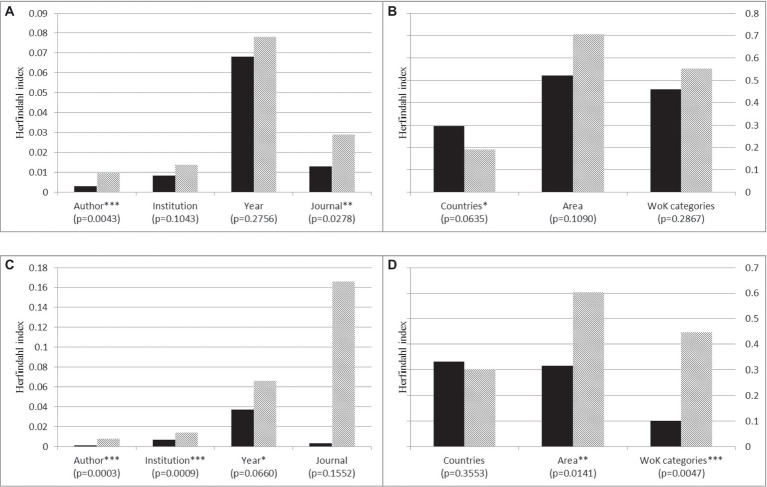
A comparison of the Herfindahl index between (I) the top five most highly cited and most highly voted papers, representing by solid black bars; and (II) the top five most highly cited but lowly voted papers, representing by grey lined bars. Figures 4A,B is the result in Management and Figures 4C,D is the result in Psychology. WoK stands for Web of Knowledge. ^*^p = 0.1; ^**^p = 0.05; ^***^p = 0.01.

To further explore what accounts for “early luck,” we examine one possible mechanism predicted by our model: Mediocre papers may receive high initial recognition or citations if they are published by authors in a favorable position in a network. If this is true, we can expect the works that cited the middling yet highly cited papers, relative to extremely highly cited ones, to be from more concentrated networks—e.g., with fewer authors, institutions, and/or journal titles. To examine this hypothesis, we first compare differences in the concentration degree between (a) the top five papers that were both highly cited and received highest numbers of votes and (b) the top five papers that were highly cited but received few votes in our survey. Specifically, we calculated the Herfindahl index for these ten selected articles^6^: $H=\overset{N}{\underset{1}{\sum }}P_{i}^{2}$ , where *H* is the index for article X, *P_i_* represents the citation share of a particular source *i* (e.g., author or journal) that cited article X, and *N* is the number of all sources that cited article X. The result is shown in Figure 4.

Our argument is supported by this concentration analysis, as Figure 4 shows. Relative to papers that received high values in both citations and votes, papers that were highly cited but received few votes are cited by more concentrated sources, including authors, institutions, and source titles, and the differences are significant.^7^ Moreover, middling yet highly cited papers in psychology also received their citations from more concentrated years, implying that citations in psychology may be more influenced by fads, gaining the peak of their citation counts and losing that momentum more quickly than the papers that received high values in both citations and votes. Note that these patterns are shared across results in both surveys, implying that the same mechanisms may still operate in psychology. A difference between the two fields, as our simulation results suggest, may be a weaker reinforcement effect in psychology than in management. In management, some middling yet highly cited papers may accumulate their high citation counts as a result of a network diffusion dynamic combined with a stronger reinforcement effect, early recognition, or luck.

**Figure 5:**
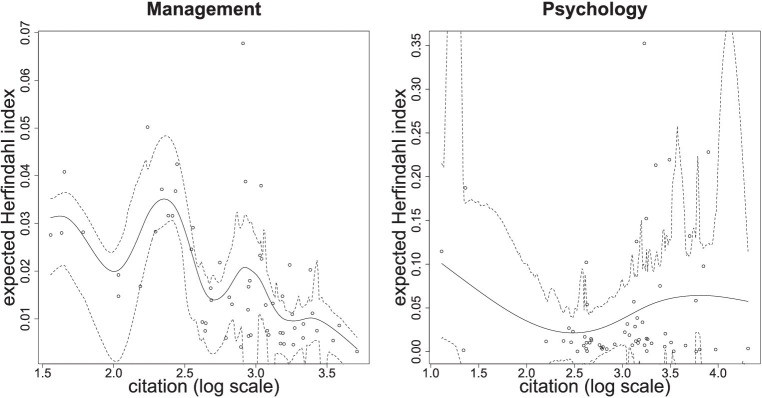
The exploratory survey results in management (left) and psychology (right). Both show how the expected Herfindahl index varies with the citation count (in log scale). The solid lines represent the best-fitted smoothing spine function to the relationship between citation and expected Herfindahl index. The dashed lines are the 95% confidence limits.

The above analysis is limited to the selected ten articles. We then further computed the Herfindahl index for all articles in our surveys based on how concentrated their citations were from the citing journals, whose sources can be identified more reliably. We were interested in how the degree of concentration (Herfindahl index) varies with citation counts. We fit cubic smoothing splines to the supplied data in both management and psychology; the results are shown on Figure 5.

The results in Figure 5 further support our “network explanation” for the middle dip and are consistent with our simulation analysis. For the results in management, the peak in the expected concentration degree coincides with the middle dip in the association between citations and impact (see Figure 4). This suggests that these moderately popular articles in management are more likely to receive their citation counts from a more limited set of sources. In contrast, the most highly cited articles in management are associated with the lowest expected Herfindahl index, implying that they receive their high recognition more evenly from different communities throughout the whole network. For the results in psychology, the association between expected concentration degree and citation is much flatter, implying that more highly cited articles do not necessarily gain their recognition only within limited communities. This is consistent with the monotonic association between votes and citation in Figure 2.

We also ran a regression analysis to examine how votes can be predicted by citation count, concentration degree, and the interaction between the two factors. The results for management are shown in Table 2. Model 1 shows that citation alone is a strong predictor for votes; highly cited articles tend to receive more votes in our survey. Model 2 shows that the concentration degree alone is also a good predictor for votes—i.e., the less concentrated the source of citations, the higher the votes are for a given article. Nevertheless, Model 3 and Model 4 suggest an interesting interaction between the two variables: a non-linear relationship between votes, citation count, and concentration degree. In particular, Model 4 suggests an inverted U-shape pattern, consistent with our results in Figures 2, 5.

**Table 2:** Regression analysis predicting survey votes in the field of management.

	Model 1	Model 2	Model 3	Model 4
Citation count	8.24** (3.88)		6.19 (4.56)	23.18** (8.78)
Herfindahl index		−267.26* (146.28)	−146.67 (170.21)	2093.80** (1014.3)
Citation count*Herfindahl index				−801.03** (357.87)
Constant	26.57** (11.10)	54.69*** (3.33)	35.04** (14.85)	−15.36 (26.69)
*R*-squared	0.08	0.06	0.09	0.17
Adjusted *R*-squared	0.06	0.04	0.05	0.12
*F*-value	4.51**	3.34*	2.61*	3.54**

*N* = 64; ^***^p < 0.01, ^**^p < 0.05, ^*^p < 0.1. Standard errors are in parentheses.

Next, we applied the same regression analysis for the data in psychology; the results are shown in Table 3. Consistent with our other results, citation count is the strongest predictor for votes in all models. While higher concentration degree predicts lower votes, unlike the results in management, the interaction between citation count and concentration degree is not significant. This suggests a less non-linear relationship for the results in psychology compared to those in management.

**Table 3:** Regression analysis predicting survey votes in the field of psychology.

	Model 1	Model 2	Model 3	Model 4
Citation count	12.17*** (3.20)		12.54*** (3.13)	13.94*** (4.22)
Herfindahl index		−47.02* (29.86)	−53.36* (26.65)	11.95 (134.53)
Citation count*Herfindahl index				−20.78 (41.95)
Constant	12.51 (9.84)	51.28*** (2.50)	13.78 (9.62)	9.51 (12.96)
R-squared	0.20	0.04	0.25	0.26
Adjusted R-squared	0.19	0.02	0.23	0.22
*F*-value	14.4***	2.48*	9.6***	6.39***

*N* = 64; ^***^p < 0.01, ^**^p < 0.05, ^*^p < 0.1. Standard errors are in parentheses.

Finally, some evidence from the voting results complements our finding that middling yet highly cited papers receive their citation counts from a more concentrated set of sources. We predict that these articles may be well known and cited within their cliques, but they may be less well known to the broader set of management academics who participated in our survey.

The voting site of All Our Ideas enables us to examine our prediction that our voting participants as a whole are less familiar with middling yet highly cited papers. Note that in Figure 1, participants were able to choose “I cannot decide” if they did not wish to choose one of the two articles in the pair comparison. If a participant chose “I cannot decide,” seven options were available, including (1) “I like both ideas,” (2) “I think both ideas are the same,” (3) “I do not know enough about either idea,” (4) “I do not like either idea,” (5) “I do not know enough about: [the idea on the left is shown],” (6) “I do not know enough about: [the idea on the right is shown],” and (7) “I just cannot decide.” In particular, options (5) and (6) enabled us to examine our prediction that participants are more likely to choose “I do not know enough about…” when encountering these middling yet highly cited papers.

The results in both management and psychology support our prediction that participants were less familiar with these middling yet highly cited papers. This suggests that these articles may not have been well known outside their cliques and hence received less recognition from our participants, who were academics in different fields of literature. In contrast, very highly cited papers were much less likely to fall into this category. In both fields, most academics recognized these outliers. This result is consistent with our concentration analysis, which suggests that the high citation counts of these middling yet highly cited papers are more likely to come from the authors’ cliques.

### Limitations and future directions

Our method, particularly the explorative surveys, have many limitations. We discuss several of them and highlight how future studies can better examine the robustness of our findings.

First, the sampling of the survey is imperfect. We used convenience sampling, and the sample size was small (680 management researchers and 943 psychology researchers), compared to the population size (tens of thousands researchers in each field). The respondents may also have been biased, particularly in management. The author who sent the survey was professionally connected to James March (the mentor of the mentor of the author), which suggests that people in management who responded to the survey may not necessarily have been representative. To strengthen the robustness of the middle-dip phenomenon between citation and impact, the survey can be replicated by sending through neutral contacts (e.g., association division representatives) with incentives to enhance response rate (e.g., vouchers for randomly drawn participants).

Second, the votes may have been driven by recognition heuristics, as academics may not necessarily know the content of all top-cited papers in their field. Our survey does allow the option of choosing “I cannot decide” when this occurs, but it does not exclude the possibility that votes were driven by participants’ knowledge about the author and/or journal instead of by the content of the paper. Future research could address this concern through a two-stage design: Voting would only occur when a participant acknowledges familiarity with the content of both articles in comparison pairs.

Another limitation is that participants only saw the seed papers plus any participant-added papers that were added before their participation, meaning that some votes were not based on the full sample. With the benefit of hindsight, we probably overestimated the number of participant-added papers, despite the crowd-sourcing design. If we had foreseen this, we would have increased the number of seed articles. It is also noteworthy that methods papers were highly cited, as most papers need a standard reference to methods. Papers included in the current pool also tended to be older ones. Future research can attenuate these concerns by introducing a two-stage survey. The first stage can solicit responses from trusted scholars to formulate a pool of articles, excluding methods papers and limiting papers older than a certain threshold. Participants from the second stage then would have access to all the papers from the end of the first phase.

Fourth, merit or quality can have multiple meanings, making connections between our survey and the chance model ambiguous. In the chance model, the merit of an object is simply a time-invariant trait drawn from certain distribution. The merit of a paper can vary greatly, depending on the context. For our purposes, merit can mean potential impact, whereas citation number captures only the realized impact. The decoupling between potential versus realized impact due to randomness interacting with a reinforcing mechanism generates a middle-dip pattern in the association between citation and merit. Yet the meaning of impact can change over time and vary across fields. Future research can ensure that the “merit” of papers is clearly defined so that participants’ answers/votes may be more commeasurable.

## General discussion

### A middle-dip version of the normative theory of luck

Many theories of luck exist. Some are based on studying the “luckiest” individuals (Wiseman, 2003) or unlucky incidents (Giustiniano et al., 2016); others study how a “serendipity mindset” could enhance the chance of important discoveries (Busch, 2022; Busch and Barkema, 2022); still others claim that most theories of luck are incoherent and by definition “wrong” (Hales, 2016).

This paper builds on a distinct theory of luck based on chance models that focuses on when higher performers indicate not just greater luck but also lower expected merit. We have reviewed three versions of a normative theory of luck and examined their predictions in the context of academic citations. The converging prediction, based on prior chance models, is that the most-cited papers are likely the luckiest and associated with lower expected merit.

Our results push back against this prediction and produce a novel version of the normative theory of luck: The performance non-monotonicity occurs not at the extremes, but in the middle range. The mechanism is that when both merit and past outcomes influence performance and when the reinforcing mechanism is strong, a high but not top level of performance entails greater uncertainty. Mediocre agents or objects can become sufficiently successful due to early good luck, plus a boost from that good luck. But a lack of merit bounds these objects’ eventual performance, such that top performance is still associated with the highest level of merit.

As Table 1 suggests, this novel version of the normative theory of luck can be considered “semi-strong”: it generates performance non-monotonicity, yet the location is not at the extreme range, as Denrell and Liu (2012) predict, but instead around high performance. The results of the chance models show one important difference in the assumption that generates the different pattern. In the current result, the reinforcing mechanism (regulated by parameter *b*) is high but fixed, mapping to the empirical context where the Matthew Effect within a field is more or less the same for all academics. If we run the model with the assumption from Denrell and Liu (2012), where the reinforcing mechanism is strong and highly variable (e.g., *b* drawn from an exponential distribution with parameter of one), the current chance model can replicate the stylized pattern of the strong version of luck, i.e., the dip occurs at the top level of performance.

Hence, our findings enrich the chance model approach and a normative theory of luck by adding a commonly observed condition for predicting when performance non-monotonicity should be expected. We discuss the implications of our findings below.

### When the Matthew effect casts doubts on quality

In studies of causal attribution processes, psychologists have argued that when an outcome has multiple possible causes, the presence of one cause casts doubt on others (Morris and Larrick, 1995). Our results fit with this normative framework of causal discounting. Moderately high citation counts can be achieved by either decent quality or initial luck (such as recognition), combined with a strong Matthew Effect. Since the presence of the Matthew Effect is well acknowledged in academia (Merton, 1968; Starbuck, 2005; Bol et al., 2018), caution may be needed in considering the quality of moderately highly cited papers.

Our chance model further suggests that a considerable discounting of moderately highly cited papers may not be unwarranted, as such papers can have lower expected quality than both their higher- and lower-cited counterparts. This is because low-quality papers are more likely to sustain moderately high citation counts when the Matthew Effect is strong (Denrell and Liu, 2021). The citation counts of high-quality papers tend to have a bimodal distribution: either exceptionally high citation counts with early recognition (i.e., initial high citations) or very low citation counts with an initial lack of recognition (i.e., initial low citations). In contrast, low-quality papers with initial recognition can ultimately gain high citation counts, but their lack of quality bounds their eventual performance despite a strong Matthew Effect.

Our survey results support this proposed mechanism. In both psychology and management, our results show an N-shaped pattern, with a “dip” in that for management and a flattening for that in psychology in the middle range. This suggests the presence of the Matthew Effect in both fields and a much stronger effect in management. Our additional analyses show that the source of the Matthew Effect may be related to network structures. Management as a field may be more fragmented than psychology, such that management academics are more likely to cite people in the same cliques, generating greater initial differences in citation behaviors and counts. This difference is then augmented by a strong Matthew Effect, partly because quality is more difficult to evaluate in a fragmented field that is short of a shared paradigm. Management academics may thus rely more on others’ choices (i.e., accumulated citation counts) to infer quality, creating a greater decoupling in the association between citation count and quality, particularly in the middle range. A strong Matthew Effect should cast doubt not only on quality, as prior research suggests. In addition, according to a “more-is-less” nuanced policy, papers receiving moderately high citation counts should receive less attention and reward, as lower-quality papers are more likely to achieve this outcome.

Our results also imply that in academia, different evaluation approaches that reflect the non-monotonic relationship shown in our results may be warranted. In institutions such as business schools, where academics from different fields are evaluated based on the same criteria, such as the journals in which they publish, citation-count analyses should be adjusted for social processes. For example, if an article garners a high citation count, this may suggest that the article is in a domain where academics publish and cite each other more than in other domains, rather than that the article is of exceptionally high quality. Moreover, our findings imply that the most likely association between citation count and quality within a school is an inverted U-shape pattern. Since extremely highly cited papers are rare, the most highly cited papers within a school are likely to be moderately highly cited papers. These papers are more likely to also reflect strong social processes rather than solely or even primarily quality. Schools should more carefully evaluate less-cited authors, as their lower citation counts may be more likely to obscure the quality of their work than that of their more highly cited counterparts.

Our results also suggest a possible solution to the problem: introducing random selection in peer review process. Our results imply that academics may be good at differentiating the best and the worst from the rest, with the former being better associated with the citation counts they deserve. This implies a solution to judging academic merit: that during the peer review process, submitted manuscripts that receive a unanimous “yes” (“no”) should (not) be published. Other manuscripts may then be published on the basis of random selection (Liu, 2021c). This solution is inspired by the recent finding that semi-random allocations of limited grant resources actually generate superior long-term outputs (Avin, 2018; Liu et al., 2020). This random process may balance the “luck factor” an article could gain from social processes.

In other words, when academics need to decide which published article to read and cite, they can rely on peer reviewers’ judgments of the best and worst articles. Such judgments may be informative of the quality of articles, but individual academics will have to rely on their own judgment when evaluating the remainder “middling” articles, because a randomly published article will likely be perceived as uninformative about quality. As a result, the citations an article receives could again be informative about quality: Citation counts would be less associated with social processes, and citation count analyses could then provide a better foundation for judging academic merit. More generally, this “random selection” proposal is consistent with recent findings suggesting that evaluations in academia should consider domain size (Radicchi et al., 2008) and that random selection can improve performance by reducing the scope of biases (Berger et al., 2020).

Our findings also suggest an opportunity. When the “middle dip” is difficult to understand, this means that some achievements may be overrated, whereas others may be undervalued. In contrast, from past chance models, our findings suggest that greater misevaluations occur around the high but not top levels of performances. Articles (and their authors) that achieved high but not top citations may be overrated; their moderate success is more likely to reflect their early luck and the resulting boost. In contrast, their lower-performing counterparts may provide a more reliable indicator of merit that may be overlooked. Schools could modify their hiring policies and search for these “hidden gems.”

### Chance model applications

The chance model approach is not mainstream in management, except for the work of March and the Carnegie Perspective. This neglect is also found in psychology, aside form in a few studies; Hilbert (2012), for example, showed how a chance model that assumes noisy information processing could account for a variety of cognitive biases.

This paper aims to highlight the valuable contribution of chance models as a non-agentic worldview that is relevant to both management and psychology. The significance of chance models and the insights they offer have been underestimated, and this research demonstrates their potential to generate novel predictions with profound implications. Emphasizing the development of chance models to account for performance differences can address the historical bias toward heroic narratives of salient agents and instead direct attention to statistical analyses, distributions, and computational methods.

Arguably, chance models could be interpreted as endorsing defeatism, since they do not provide explicit causal explanations or immediate pragmatic implications. Telling students that performance differences can result from luck could be demotivational. By contrast, we propose that chance models offer causal explanations when their theoretical mechanisms produce predictions that closely approximate the empirical regularities they aim to explain. By systematically simulating counterfactual histories, management scholars and practitioners can extract more rigorous lessons from successes and failures, which often represent unique instances. Moreover, a deeper understanding of the role luck plays in performance can help individuals in management overcome the illusion of complete control, leading to improved performances within their control and better preparedness for unpredictable situations.

Given that the realized history is just one potential outcome drawn from a distribution of numerous possible histories, it becomes imperative for scholars to take chance models and the alternative histories they generate seriously. Relying solely on sophisticated regression methods may not rescue the biased lessons inherent in the realized history. Embracing chance models enables a more nuanced view of historical events that promotes a richer understanding of socio-behavioral dynamics and provides valuable insights for informed decision-making.

### When the wisdom of the crowd fails

The idea of the wisdom of the crowd—that aggregating the independent estimates of a diversified group of people produces more accurate estimates than those produced by individuals—implies that popular choice is informative (Page, 2008). Prior studies have suggested several mechanisms that can undermine the wisdom of the crowd (Lorenz et al., 2011). Information about others’ choices is likely to homogenize people’s expressed beliefs in two ways. The informational aspect of social influence suggests that people may hold back private beliefs and sample popular choices because they believe others have superior information (Bikhchandani et al., 1992; Salganik et al., 2006). The normative aspect of social influence suggests that people may abandon private beliefs and conform to others’ beliefs because they feel uncomfortable acting against the crowd (Asch, 1951; Kuran, 1997). Thus, rich-get-richer dynamics, or the Matthew Effect, can undermine the wisdom of the crowd because popular choices may reflect self-reinforcing expressed beliefs decoupled from actual private beliefs in the presence of social influence. The implication is that more popular objects can be worse when social influence is strong.

Our results suggest that the crowd can be wise globally but foolish locally when the Matthew Effect is present but bounded by structure. Many mechanisms that generate conformity operate through networks and are sensitive to the overall structure of connections. We have demonstrated that the local nature of social influence implies that conformity is likely bounded locally and operates only within cliques. Beyond local networks, normative social influence is weakened. People may be aware of popular objects (e.g., highly cited papers) due to informational aspects of social influence and rely on their own judgment when deciding whether to adopt objects (e.g., citing the papers or not). The implication is that local popularity—the crowd’s choice within a clique—is likely to reflect situations where social influence collapses the wisdom of the crowd. In contrast, global popularity—the crowd’s choice throughout different networks—is more likely to reflect situations where the wisdom of the crowd does trump social influence. Overall, results from our surveys, the chance model we then developed, and the analyses we iterate against the survey data generalize across conditions under which the crowd is wise or foolish.

### Implications for diversity

Our findings, which build on the broader perspectives offered by the chance model, pose some discussion points that may be particularly timely as society grapples with issues relating to diversity, equity, and inclusion. Further, the implications of our work may be particularly relevant for academia, an institution that has come under scrutiny for how marginalized groups have remained underrepresented at every level, from students at elite universities to tenured professors who hold nearly unparalleled job security.

Historically, advantaged groups (e.g., men, White individuals) have been more privileged at each life milestone and round of professional evaluation (and rewards), ultimately accumulating important leadership roles that then position them to be more likely to perpetuate the same class structures through multiple mechanisms, including homophily in hiring and the transmission of intergenerational wealth. Though recent initiatives have made strides in remedying the lack of minority representation and the associated socioeconomic and health consequences that have disproportionately impacted minorities, one result may be backlash from those in advantaged groups who question the processes through which greater diversity was achieved. The polarized political landscape in the United States is one reflection of these competing narratives about what should be considered merit and fair allocation of valued rewards.

We return to one implication of our results, namely the idea that academic papers with low citation counts may need extra consideration, as citation counts may reflect a lack of initial recognition rather than a lack of quality. Although the argument that papers with fewer citations (or less recognition) may be of higher quality than those with moderately high citations (or more recognition) extrapolates beyond the limits of what our models would suggest, we propose that the underlying premise may still be informative in nudging people to consider whether a surface-level evaluation of present output or performance is sufficient. This sets aside longstanding issues with evaluation metrics, such as whether there is correspondence between HR processes and eventual hiring decisions, or whether impressions or scores at the point of hiring ultimately predict later job performance. Instead, we propose that by thoughtfully deliberating the pathway candidates traversed to arrive at their current level of performance, a journey that may have been riddled with chance events both good and bad, there may be promising avenues for cultivating more sustainable and long-term quality performance.

We hope some aspects of our model may spur fruitful conversations. For example, a fresh look at the significant role of chance and initial successes or failures, which are then magnified through reinforcement, may offer new perspectives for decision-makers involved in crafting policies aimed at establishing fair systems of evaluation and compensation. These efforts may buffer against the systematic discounting of lower initial or current performance—which is often associated with marginalized groups—and foster organizational cultures that value not just the most accessible quantitative performance data but also data about the range of pathways to achievement.

## Data availability statement

The codes and the data supporting the conclusions of this article will be made available by the authors, without undue reservation.

## Author contributions

All authors listed have made a substantial, direct, and intellectual contribution to the work and approved it for publication.

## Footnotes

^1^The Matthew Effect was coined by Robert Merton in his 1968 *Science* paper. The term is inspired by a paragraph in the *Gospel of Matthew*: “For to every one who has will more be given, and he will have abundance; but from him who has not, even what he has will be taken away.” Merton used these sentiments to describe the unequal fame gained in incidents of simultaneous discoveries, with one taking all the credit while the other becomes obsolete. One example surrounds the development of calculus: The divergence between the contributions of Sir Isaac Newton and Gottfried Wilhelm Leibniz. While both mathematicians made substantial advancements in the field, Newton is commonly attributed with the discovery of calculus, primarily owing to his influential position within the scientific community and his association with the English-speaking world. Leibniz, on the other hand, presented a distinct formulation of calculus that bears closer resemblance to its contemporary usage. Despite the inherent intricacies of the attribution process, Newton’s prominent stature and the dissemination of his work in the English language have contributed to the prevailing perception of him as the primary progenitor of calculus, thus shaping the historical narrative surrounding this fundamental mathematical discipline.

^2^We intentionally use “which article is the best” to avoid a demand effect from the participants—selecting the answers (articles) based on their inference of what the researchers want to find or consider appropriate. We acknowledge that this choice is imperfect because “best” does not necessarily mean “greater impact.” Yet the dictionary definition of “best” is “of the most excellent or desirable type or quality,” (Oxford English Dictionary) which is generic enough to serves our purpose.

^3^A limitation is that participants only saw the seed papers plus any participant-added papers that were added before their participation.

^4^We applied the cubic smoothing spline with a loss function that obtained the smoothest function without overfitting the data.

^5^The N-shaped pattern is not driven by the participant-added articles. Additional analyses show that papers in the middle dip (highly cited yet lowly voted) are mostly seed articles from certain fields (e.g., entrepreneurship).

^6^This number is selected arbitrarily for illustration purpose. We apply this analysis to all articles with the results reported below.

^7^The only variable that shows an opposite concentration pattern is “Countries.” This result may still support our argument in a general sense, considering that most extremely highly cited papers are primarily published by authors and journals based in the United States. One might imagine a handful of academics outside the United States could group together over time and cite each other frequently to gain legitimacy or out of greater familiarity. This finding suggests an interesting topic for future research in the sociology of science.

## Appendix A: Survey invitation email text

Title: The BEST Management [Psychology] journal articles – Have your say!

Greetings,

We develop an on-line voting tool (using ‘All of Our Ideas’) for finding out the best journal articles published in Management [Psychology]. We have selected 45 [60] articles in the voting pool (the top 3 most cited articles in the 15 [20] Management [Psychology] journals, see the list below). Feel free to add your favorite articles to the pool (as long as it’s published in one of the selected 15 [20] journals)!

Here is what you need to do to express your views on the best article in Management [Psychology]:

First, log on [link] and begin voting! You will be presented with randomly selected pair-wise comparisons. Keep voting for as long as you like!

Second, feel free to select ‘I cannot decide’ in cases such as you are not

familiar with the article(s) in the pair or you do not believe there is significant difference between them.

Third, forward this email or the link to your colleagues and let them have their say on this issue!

We thank you for your attention and participation!

==Journal List==.

1 Academy of Management Journal (AMJ)

2 Academy of Management Review (AMR)

3 Administrative Science Quarterly (ASQ)

4 California Management Review (CMR)

5 Harvard Business Review (HBR)

6 Human Resource Management (HRM)

7 Journal of Applied Psychology (JAP)

8 Journal of Business Venturing (JBV)

9 Journal of International Business Studies (JIBS)

10 Journal of Management Studies (JMS)

11 Management Science (MS)

12 Organization Science (OS)

13 Organizational Behavior & Human Decision Processes (OBHDP) (formerly Organizational Behavior and Human Performance)

14 Sloan Management Review (SMR)

15 Strategic Management Journal (SMJ).

[For Psychology].

==Journal List==

1 BEHAV BRAIN SCI

2 ANNU REV PSYCHOL

3 PSYCHOL BULL

4 TRENDS COGN SCI

5 ANNU REV CLIN PSYCHO

6 PSYCHOL REV

7 ADV EXP SOC PSYCHOL

8 PERS SOC PSYCHOL REV

9 AM PSYCHOL

10 MONOGR SOC RES CHILD

11 CLIN PSYCHOL REV

12 J PERS SOC PSYCHOL

13 PERSPECT PSYCHOL SCI

14 PSYCHOL MEN MASCULIN

15 DEV PSYCHOPATHOL

16 J EXP PSYCHOL GEN

17 J APPL PSYCHOL

18 J ABNORM PSYCHOL

19 PERS PSYCHOL

20 PSYCHOL SCI
